# A review of paper-based advance care planning aids

**DOI:** 10.1186/s12904-018-0298-0

**Published:** 2018-03-27

**Authors:** John F. P. Bridges, Thomas Lynch, Anne L. R. Schuster, Norah L. Crossnohere, Katherine Clegg Smith, Rebecca A. Aslakson

**Affiliations:** 1 Department of Health Policy and Management, The Johns Hopkins Bloomberg School of Public Health, Baltimore, MD USA; 2 Department of Anesthesiology and Critical Care Medicine, The Johns Hopkins School of Medicine, Baltimore, MD USA; 3 Department of Health, Behavior and Society, The Johns Hopkins Bloomberg School of Public Health, Baltimore, MD USA

**Keywords:** Advance care planning, Patient decision-making, Patient-centered care, Decision aid

## Abstract

**Background:**

Advance care planning (ACP) aids can help prepare patients, family members, and physicians for in-the-moment medical decision-making. We wished to describe the content and approach of paper-based ACP aids in order to characterize existing aids and inform the development of a new ACP aid.

**Methods:**

Paper-based ACP aids were identified through an environmental scan and screened for eligibility. ACP conceptual frameworks and data were gathered via stakeholder engagement and used to inform the coding framework that two investigators used to independently code each aid. A directed content analysis was conducted on these eligible aids. Aids were categorized through a deliberative process with an investigator abstracting general information for each aid.

**Results:**

Fifteen aids met the eligibility criteria. They ranged in length from 6 to 78 pages with the average aid written at an eighth-grade reading level. The content analysis revealed that many aids encouraged choosing a surrogate decision maker and informed users about legal medical documents. Fewer than half of the aids facilitated patient clarification of values regarding quality of life issues. The authors identified and termed the following three categories of aids: informative; semi-action oriented; and action-oriented. It was often unclear whether patients contributed to the development or testing of the ACP aids reviewed.

**Conclusions:**

Most existing paper-based ACP aids address legal matters such as completing an advance directive. Only a minority elicited patient values and it was unclear whether any were developed in partnership with patients. Future development of ACP aids should account for patient preferences with a goal of supporting in-the-moment medical decision-making.

## Background

Advance care planning (ACP) offers individuals the opportunity to clarify their health care goals, concerns, and wishes in preparation for situations where they may be unable to make their own decisions [1]. While ACP has traditionally encouraged patients to complete advance directives [2–4], today the focus of ACP has been broadened to include complex conversations between a patient, their alternative decision maker, and their physician [5]. Taking such steps is intended to prepare patients and their alternative decision makers to make in-the-moment, values-based choices that better account for patients’ current and evolving clinical situations and reflect patients’ and alternative decision makers’ goals and needs [6].

ACP decision aids are designed to facilitate deliberation of preference-based medical decisions, and may benefit patients and alternative decision makers as they prepare to make in-the-moment medical decisions [7, 8]. A recent environmental scan identified ACP aids in a range of formats (paper, video, audio, and web) that could potentially support the process of ACP [9]. A systematic review [10] following this scan determined that out of the 30 unique aids examined, the majority were either video-based (*n* = 15), and have been since explored in more detail [11], or were paper-based (*n* = 10). Although the preferred aid format might vary based upon characteristics of the patient, provider, and clinical setting [12, 13], paper-based aids represent a particularly powerful vehicle for ACP given that they are an already established and popular form of decision aid [10], and are more accessible to patients and require fewer resources to administer than video or web-based aids [12].

While it would be practical for most individuals to initiate ACP prior to becoming ill, evidence indicates that 70% of the general US population do not have advance care plans [14] in spite of nation-wide efforts to promote ACP [15–17]. Even when ACP is addressed, finding an aid that best matches the patient, physician, and circumstances surrounding a patient’s illness can be a challenging feat given the absence of comparative effectiveness data regarding ACP aid formats [10]. With this information in mind, a directed content analysis [18] of existing paper-based ACP aids was conducted in order to better understand the diversity of paper-based aids. The research team undertook this content analysis as a part of a larger Patient-Centered Outcomes Research Institute-funded project to develop an ACP aid.

The purpose of this study was to describe and analyze the content and style of paper-based ACP aids. The study further sought to categorize the aids so as to provide patients and providers with more information that might help to inform their choice of an ACP aid. This work is also anticipated to provide insight into the ways that ACP aids could possibly be designed to further meet the needs of patients, their families, and clinicians as they prepare for possible in-the-moment medical decision-making. Finally, this work could help inform the development of a new ACP tool.

## Methods

Contributing to the environmental scan [9] to identify and assess ACP aids, this study adopted a two-stage approach: 1) a one-day stakeholder summit; and 2) a directed content analysis of paper-based ACP aids which served to extend prior research by further describing phenomena of ACP aids’ substance, focus, and structure [18].

### Stakeholder summit

A stakeholder summit was convened to engage stakeholders and identify relevant elements of paper-based ACP aids. Stakeholders (*n* = 14) included two patient family advocates, three surgeons, one internist, two health services researchers, four palliative care clinicians and/or researchers, one patient-centered outcomes researcher, and one patient safety and quality researcher. A diverse group of stakeholders were deliberately engaged so as to include a wide-range of perspectives in the discussion surrounding decision aids. Invited participants from each of these stakeholder groups were selected based upon their interest in and experiences with advance care planning, as well as based upon previous work with the research team. Using design-thinking techniques [19, 20], the stakeholders identified key elements of a representative sample of seven paper-based ACP aids. These techniques included activities such as brainstorming ideas for ACP aids, dot voting on preferred attributes of aids, and group discussion about the benefits and risks of aids so as to engage stakeholders to provide opinions and insight that might ultimately help the research team develop a new ACP aid. The diverse sample of seven aids were purposefully selected by the research team for discussion at the summit as they collectively demonstrated how ACP aids vary in terms of structure, length, presentation style, and content. The findings from the stakeholder summit were largely complementary to the content analysis and helped the research team elicit general user feedback on patient, caregiver, and other stakeholder’s preferences and priorities for ACP aids.

### Paper-based ACP aid content analysis

#### Search and selection of paper-based ACP aids

Paper-based ACP aids were identified through the following components of the environmental scan [9]: a systematic review [10], a grey literature review of un-published or in-progress data [9], and key informant interviews [21]. Paper-based aids were included in this study if they met the following inclusion criteria: addressed ACP; existed as a paper handout, pamphlet, and/or pdf file; were written in English; and addressed at least one aspect of a decision aid as defined by the Cochrane review of decision aids [8] insofar as they provided methods to clarify values and/or information about treatment options and outcomes. State-sanctioned living wills and individually-generated advance directive forms were excluded from the study.

#### Abstraction of data

Several months after completing the environmental scan, one researcher used a structured abstraction tool to abstract general information and basic data about each of the paper-based ACP aids and another researcher confirmed the accuracy of this abstraction data. The information abstracted included characteristics about the document such as its length, word count, the producer’s name and the country where the producer was located.

In addition, researchers abstracted information about elements pertaining to the accessibility of printed materials [22, 23]. These elements included readability based on the Flesch-Kincaid Grade reading level [24] using Microsoft Word’s (version 10, Microsoft Corp., Redmond, WA) readability test [25], and presentation characteristics such as strategies to engage the user (i.e., question-answer formats) and improve page layout (i.e., bullet points, short paragraphs)^.^ [22, 23].

As multiple aids could be classified as decision support tools, the investigators used a component of the International Patient Decision Aid Standards (IPDAS) checklist [26] to abstract information about the paper-based ACP aid development process including whether it: (1) identified developers’ credentials and/or qualifications; (2) identified what users potentially need in order to discuss their options; (3) incorporated patient and/or health care providers in the development process; and (4) involved field testing of the aid. For each of the four criteria, a paper-based ACP aid was assigned a 0 if did not fulfill that criterion or a 1 if it fulfilled that criterion; these scores were then summed for a total score out of 4.

#### Coding of ACP content

Consistent with a directed content analysis approach [18] and methods utilized in a similar study [27], researchers identified initial coding categories based on existing ACP conceptual frameworks [2–4, 6] stakeholder engagement findings, and a review of three paper-based ACP aids. Two research team members (VP, TL) independently coded the paper-based ACP aids using the predetermined codes and tracked results using a spreadsheet developed in Excel (version 2010, Microsoft Corp., Redmond, WA). Researchers noted data that could not be coded with the initial codes and later determined if the data represented a new category or possibly a subcategory of an existing code [18]. Discordance was resolved by mutual consensus and through engaging a third reviewer when required (ALRS).

Content was coded as pertaining to the clarification of patient priorities and values in general, and specifically in relation to states “worse than death” and patients’ preference for how much leeway a surrogate decision-maker should possess when making decisions on behalf of the patient [6]. Codes were developed for content relating to naming a surrogate decision-maker and providing guidance to surrogates on their roles and responsibilities [2–4, 6]. Six ACP content categories that were ultimately delineated through the content analysis: ACP definitions, values clarification, ACP conversations, surrogate decision-maker, survival odds or statistics, and form descriptions.

Additionally, researchers coded information that related to patient discussions about ACP and support for expressing their wishes, including examples of potential ways to start ACP conversations. Finally, the framework also addressed content about legal medical documents and ACP resources, and decisions about after-death issues (i.e., organ donation and burial) [2–4, 6].

#### Analysis

The content was described and analyzed qualitatively [18, 27]. Codes were aggregated across the aids, summarized descriptively, counted, and depicted graphically in order to enable visual comprehension of the content [18]. All aids were categorized [28] and prioritized using a deliberative process involving multiple investigators (VP, TL, ALRS, JFPB). Specifically, the researchers reviewed the aids and grouped them according to their concordance in content delivery. The groups were then labeled based on their delivery approach.

## Results

### Stakeholder summit

In brief, the stakeholders did not recommend the use of content or images that framed ACP from an end-of-life perspective; they felt that end-of-life framing for ACP was “off putting” and could deter patients from engaging with ACP early in their disease course. Stakeholders identified support for patients in choosing a surrogate, opportunities to identify personal priorities, and assistance in enabling patients to imagine states “worse than death.”

### Paper-based ACP content analysis

**Fig. 1:**
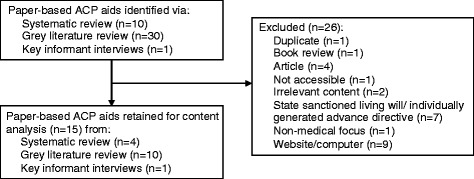
Selection results for paper based ACP aids

Of 41 paper-based ACP aids identified, 15 met the inclusion criteria (Fig. 1). Ten of these aids were identified by the grey literature search, 4 were identified from the systematic review, and 1 was identified by a key informant. Table 1 provides a summary of data abstracted from the aids while Table 2 presents descriptive details about each one. The documents were primarily produced by organizations in the USA (14/15; all aids except 13) and averaged 25 pages in length (ranging from 6 to 78 pages). The majority of tools used different color fonts (13/15; all aids except 10, 13), and did not include pictures (8/15; aids 1-3, 9-11, 13-15). On average, the aids were written at an eighth grade reading level (ranging from the fifth grade to the eleventh grade).

**Table 1:** Summary of data abstracted from paper-based ACP aids (*n* = 15)

	Range	Mean	Percent
Basic data			
Page length	6 - 78	25	–
Word count	1212-19,122	6825	–
Country where produced	US & Can*1*	–	–
Accessibility elements			
Readability based on Flesch-Kincaid Grade Level	5.5 – 11.7	8.5	–
Use of color	–	–	86
Use of photographs with actual people	–	–	46
Layout (bullet points, short paragraphs, white space)	–	–	67
Question-answer formats (open-ended questions, Likert scales, quizzes)	–	–	67
IPDAS development process			
Identified developers’ credentials and/or qualifications	–	–	67
Identified elements users need to discuss their ACP options	–	–	0
Involved patients and/or health care providers in development	–	–	7
Included users in field testing of the paper-based ACP aid	–	–	0

**Table 2:** General information about paper ACP aids

Title (assigned number)	Origin	Org.	# pages	# words	Read level^a^	Cat.	Real pictures	Color	Dev. Score
Your Conversation Starter Kit (1)	USA	IHI	10	1977	5.5	AO		✔	0
Consumer’s Tool Kit for Health Care Advance Planning (2)	USA	ABA	26	7362	8.4	AO		✔	1
Your Life Your Choices (3)	USA	VA	53	18,937	5.5	AO		✔	1
Planning for Future Health Care Decisions My Way (4)	USA	VA	78	19,122	7.1	AO	✔	✔	1
Caring Conversations: Making Your Healthcare Wishes Known (5)	USA	CPB	16	6210	9.4	AO	✔	✔	2
How to Talk to Your Doctor (6)	USA	IHI	9	2314	7.5	SA	✔	✔	0
10 Conversations to Plan for Aging with Dignity & Independence (7)	USA	TSF	7	1917	10.4	SA	✔	✔	1
Advance Care Planning (8)	USA	HFSA	28	2574	11.7	SA	✔	✔	1
Five Wishes (9)	USA	AD	11	1212	7.4	SA		✔	1
The Medical Directive (10)	USA	Res	7	2494	8.8	SA			1
Looking Ahead: Choices for Medical Care When You’re Seriously Ill (11)	USA	IMDF	59	13,703	8.4	In		✔	1
If Talking Is So Important Why Is It So Hard? (12)	USA	NHPCO	12	4948	6.8	In	✔	✔	1
Living Will (13)	CA	–	24	10,005	10.9	In			1
End-of-Life Decisions (14)	USA	CC	23	8103	10.5	In		✔	0
Advance Directive: Planning Ahead (15)	USA	KP	6	1501	9.2	In	✔	✔	0

^a^Flesh-Kincaid Grade Level, *Org* Organization that produced video, *USA* United States of America, *Can* Canada, *Cat* Category AO: action oriented, *SA* Semi-action oriented, *In* informative, *IHI* Institute for Healthcare Improvement, *ABA* American Bar Association, *VA* Department of Veteran Affairs, *CPB* Center for Practical Bioethics, *IHI* Institute for Healthcare Improvement, *TSF* The Scan Foundation, *HFSA* Heart Failure Society of America, *AD* Aging with Dignity, *IMDF* Informed Medical Decision Foundation, *CC* Caring Connection, *KP* Kaiser Permanente, *Dev score* Development process score according to one component of IPDAS checklist

Investigators identified three categories of aids (Table 2), which were termed action-oriented (5/15; aids 1-5), semi-action oriented (5/15; aids 6-10), and informative (5/15; aids 11-15). Semi-action and action-oriented components included question-answer features such as quizzes, multiple choice and open-ended questions, and questions with Likert-type scales. Action-oriented aids additionally offered patients the opportunity to reflect, synthesize their answers, and draw conclusions or insight from their perspectives, whereas the semi-action oriented aids did not include these types of engagement strategies. The action-oriented aids were written on average at a seventh grade reading level, while the semi-action oriented and informative aids were written on average at a ninth grade reading level. Informative aids did not attempt to engage the reader and rather offered educational information surrounding ACP.

In terms of the paper-based ACP development process, the majority (10/15; aids 2-4, 7-13) of the aids scored a one out of four where all of these tools identified the developers’ credentials and/or qualifications. One aid (1/15; aid 5) met two of the four criteria by additionally seeking input from healthcare providers during the development process. It was unclear whether any of the tools had been developed, field-tested, or informed by patient feedback.

While not all aids included content from each of the six APC content categories, every aid contained content across more than one category. The following sections describe each of these content categories in more detail.

### ACP definitions

Over half of the aids (9/15; aids 3-8, 13-15) explicitly defined ACP. Among these aids, some or all of the following topics were defined as a component of ACP (Table 3): identifying the patient’s care goals; communicating with loved ones and health care providers; choosing a surrogate decision maker; and completing an advance directive. Six aids specifically outlined a process for completing ACP in order to enable patients to express their wishes and/or reexamine them. Most commonly (5/6; aids 1-3, 5) the process included the “why, when, where, and to whom” patients should express their wishes.

**Table 3:** Content categories and illustrative paper-based ACP aid excerpts

Category	Representative quotation or excerpt (paper based ACP aid #)
1. ACP definitions	“Advance care planning is a step-by-step process to help you plan for medical decisions in your future.” (4) “Advance care planning is the ongoing process of discussing values and goals of care, determining and/or executing treatment directives and appointing someone to speak for you when you cannot speak for yourself.” (5)
2. Values clarification	“What matters to me is ________. Start by thinking about what’s most important to you. What do you value most? What can you not imagine living without?” (1) “What do you most value about your physical or mental well-being? For example, do you most love to be outdoors? To be able to read or listen to music? To be aware of your surroundings and who is with you?” (2)
3. ACP conversations	“Perhaps the single most important step in ACP is talking about your wishes with whom might be called upon to speak for you. There is no ‘right way’ to start this conversation. Nor is there a ‘right’ time. The best thing to do is make time and get started.” (3) “There are many events and openings that can help you get started [with the conversation].Conversation triggers include: the death of a friend or colleague; newspaper articles about illness and funerals; movies, sermons…” (12)
4. Decision-maker selection	“On a scale from ‘I want my loved ones to do exactly what I’ve said’ to ‘I want my loved ones to do what brings them peace even if it goes against what I’ve said’, how involved do you want your loved ones to be?” (1) “Picking the right person to be your health care agent. Choose someone who knows you very well, cares about you, and who can make difficult decisions…” (9)
5. Survival odds or statistics	“Would you be willing to endure severe side effects if the chance that you would regain your current health was: high [over 80%]; moderate [50%]; low [20%]; very low [less than 2%]; very, very low [less than 1 in 1000]” (2) “CPR Pros & Cons. Pros: CPR can save lives, especially when it’s given to a young, healthy person right after cardiac arrest….Cons: CPR is often unsuccessful, especially when it’s given to someone who has a very serious or incurable disease. Most hospitalized patients who get CPR don’t survive to leave the hospital….” (4)
6. Form descriptions	“Through a legal document known as a Durable Power of Attorney for Healthcare Decisions, you can designate a person to make health care decisions on your behalf should the need arise.” (7) “The Medical Directive allows you to record your wishes regarding various types of medical treatments in several representative situations so that your desires can be respected.” (10)

### Values clarification

The paper-based ACP aids offered patients a variety of opportunities to contemplate and express their values about different aspects of ACP. Nearly half the tools (6/15; aids 1, 3-5, 9, 14) contained exercises that enabled clarification of priorities and values regarding quality of life with statements such as “*I want to live as long as I can no matter what.*” Four tools (aids 1, 3-5) enabled readers to clarify their role in making health care decisions and contemplation of feelings about potential choices such as “*I want to know the basics*” or “*I want to know as much as I can.*” Seven tools (aids 2-5, 9, 10, 13) asked patients to imagine different medical or health situations that they could find themselves in and asked patients to specify their advance treatment preferences.

### ACP conversations

**Fig. 2:**
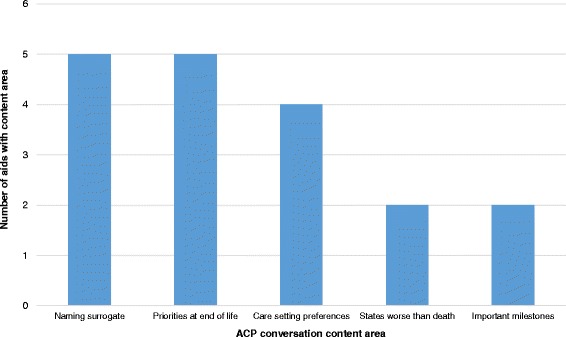
ACP paper-based aid conversation content area

In support of communicating patients’ medical wishes, ten aids contained information about ways for patients to start a conversation relating to ACP (aids 1-8, 12, 13). The content ranged from example phrases, vignettes, and settings to potential formats (i.e., letters, audio-recordings) for initiating the ACP conversation (if not in person) and tips for dealing with resistance to that conversation. Beyond offering information about how to start a conversation about ACP, a majority of aids (11/15) also provided information about conversation talking points (Fig. 2) such as “who will be your surrogate decision maker” (5/15; aids 1-5), the patient’s preferred role in medical decision-making (4/15; aids 1, 3, 7, 9), states “worse than death” (2/15; aids 2, 3), milestones that the patient would like to attain (2/15; aids 1, 6), and what matters most during the last phase of life (5/15; aids 1, 3, 4, 6, 8, 12).

### Surrogate decision-maker

Of these paper-based aids, one third (5/15; aids 6, 10, 11, 13, 15) did not advocate for or provide assistance with choosing a surrogate. Among the ten that did discuss choosing a surrogate, the type of support ranged from: (i) describing the importance of having a surrogate (8/10; aids 1-5, 7, 8, 14); (ii) telling personal stories about choosing a surrogate (4/10; aids 3, 4, 12, 14); (iii) providing the opportunity to consider different reasons why a person would make a suitable surrogate and comparing individuals across those dimensions (5/10; aids 2-5, 9). Six of the ten aids also offered patients the opportunity to express how much leeway they were comfortable in granting to surrogates when they were making decisions on the patient’s behalf (aids 1-5, 9). Statements within the aids such as “*I want my loved ones to do exactly what I’ve said*” or “*I want my loved ones to do what brings them peace even if it goes against what I’ve said*” illustrated the concept of surrogate leeway in decision-making.

Across all of the paper-based ACP aids, nearly half of the aids (7/15; aids 1, 2, 4-6, 13, 14) contained content aimed at surrogate decision makers about their roles and responsibility. Additionally, three of the aids provided guidance on making decisions for someone else (aids 2, 5, 6). Interestingly, some of the aids that did not provide support for designating an alternative decision maker did include information specifically for the surrogate decision maker (aids 6, 13).

### Survival odds or statistics

Two aids presented information about the odds of survival and presented information about survival statistics for particular treatments (such as the chances of recovering from CPR; aids 2, 3). Furthermore, five aids provided users with a description or depiction of different illness trajectories (i.e., slow decline, steady decline with periodic crises, lingering expected death, sudden death; aids 3-5, 10, 11).

### Form descriptions

Almost all of the aids examined in the content analysis (14/15; all aids except 7) provided information relating to legal and non-legal medical documents. The information included topics such as: (i) explanations of legal medical documents such as advance directives and durable power of attorney of health care; (ii) instructions on how to complete these forms; (iii) treatment definitions that could be of use when completing legal medical documents; (iv) additional resources for where patients could obtain more information; and (v) reminders about when to review and revise medical documents. A minority of aids (2/15; aids 3, 13) also provided opportunities for users to complete wallet cards that specify emergency contact information. The purpose of these wallet cards were such that emergency or medical personal might reference these cards to find the contact information of a surrogate decision maker in the event that the cardholder was unable to speak for her- or himself.

## Discussion

The successful implementation of ACP aids could potentially improve the provision of medical care, especially for patients who are more likely to lose medical decision-making capacity.

Indeed, the 2015 landmark Institute of Medicine (IOM) report “Dying in America” specifically advocates for more ACP among all patients and their family members [29]. Our study identified three categories of paper-based ACP aids and found that they vary in their ability to support deliberate decision-making—an element that is important to making preference-based decisions [7]. The action-oriented tools, which comprised one third of the aids, encouraged active participation in the process of ACP through questions that stimulated contemplation and synthesis of patients’ thoughts in order to help them clarify and identify their values. Among the action-oriented aids, a few also provided exercises that encouraged participants not only to identify their values and preferences, but also to make meaning of them through active reflection. ACP aids that support meaningful deliberation and participation in decision-making, as opposed to solely informative aids, may enable participants to more fully immerse themselves in ACP and to weigh their own unique goals and preferences when they are considering ACP.

While the reviewed aids were both detailed and informative, the reading level of aids may limit their efficacy. As approximately half of all adults in the U.S. read at or below an eighth grade reading level [30], the IOM recommends that health materials be written at a sixth-to-eighth grade level in order to facilitate better access to information and active participation in decision-making processes [31]; both of these outcomes can be even further negatively impacted by limited health literacy [32]. While action-oriented aids were generally written within the recommended grade reading levels, the semi-action oriented and informative tools were generally written above the IOMs recommended reading levels. The proportion of aids written at advanced-grade levels mirrors previous research that found most advance directives have been written at the twelfth-grade reading level [33].

The content analysis revealed that many stakeholder-identified key components of ACP aids were missing from these paper-based aids. For instance, stakeholders disliked framing ACP from an end-of-life perspective because they felt that this focus could deter patients from engaging with ACP early in their disease course. Yet, most of the examined aids adopted an end-of-life perspective by focusing on legal medical forms and providing information about advance treatment preferences specifically for end-of-life scenarios. Moreover, as some evidence suggests that legal medical documents about advance treatment preferences are limited in terms of their ability to actually inform medical care for patients who cannot speak for themselves [6, 34–37], we need new and innovative ways to expand the focus of ACP aids beyond end-of-life perspectives. As stated by a key informant in a previous study, decision aids need to “pull out of the end-of-life rut and get into the quality of life groove,” [21].

ACP conceptual frameworks [2–4, 6] and our stakeholder engagement results both stress the importance of providing information and tools to help people choose a surrogate decision maker. While most aids provided information about the legal importance of naming a surrogate, only a few of them provided additional guidance and support in selecting someone who could potentially be best suited to the role. The more structured aids included exercises for patients to identify, compare, and select potential surrogates based on qualities that would make them well-suited to the role and responsibilities of being a surrogate. Patient and family member input would provide significant, “real life” and meaningful insight into how to select a potential surrogate decision maker and how to further discuss care goals and treatment preferences with that individual. Frontline care providers can also facilitate this process by: (i) proactively asking patients to name a surrogate decision maker, particularly at early stages of serious illness, and (ii) advocating for the patient to have an ACP discussion with their surrogate, possibly with that discussion facilitated via an existing paper, video, or other type of ACP aid [10].

Interpretation of these results should take into consideration the study’s limitations. Researchers might have neglected to include useful measures in the development of the coding framework. It was, however, based on components that have been identified previously as meaningful to advance care planning [6] and to the production of high-quality aids [7, 26]. Additionally, the sample of aids reviewed in this study may not be representative of all such paper-based tools, though this study analyzed all of the instruments identified through a recently conducted environmental scan [9] that included key informant interviews [21]. The abstraction of information from the aids may also be subject to differing interpretation, although the researchers utilized standard methodologies to refine and resolve misunderstanding of the concepts being abstracted. Further, inconsistencies in how the aids report publication information such as year, as well as the frequent updating of the aids by their distributing healthcare organizations, poses a limitation to accessing past versions of the aids and replicating the results of the content analysis. Finally, comparison of findings from the content analysis with the results from our stakeholder engagement process may have resulted in an incomplete set of guidelines by which to gauge the likely benefit of the aids.

Patient involvement in the development of these ACP tools was unclear. It is important to gather further stakeholder perspectives and to specifically engage patients and other key stakeholders in the development process of any ACP aid, especially considering that ACP aids should facilitate patient, healthcare professional, and community involvement in decision-making [38]. Subsequent work and research is needed to identify patient preferences and acceptance of aid characteristics in relation to ACP.

## Conclusion

This study identified a need for paper-based aids that support and empower patients to initiate meaningful ACP. All examined aids presented ACP solely within an end-of-life framing. The findings suggest that to meet patients’ needs and create patient-centered ACP aids, active patient involvement is a necessary, and often missing, component and ACP aids need to explore framing beyond specific end-of-life care situations. By identifying and classifying different categories of paper-based ACP aids, the study provides a useful framework that clinicians might use to help inform the selection of an ACP aid that is most appropriate to their patients and to alternative decision makers. Additionally, these findings have helped guide the development of a new ACP aid [39–41], and have provided a framework by which future ACP aids might be measured.

## Abbreviation

ACP: Advance care planning
